# Atypical Presentation of Congenital Sucking Blister of the Lip: A Diagnostic Challenge

**DOI:** 10.7759/cureus.112816

**Published:** 2026-07-16

**Authors:** Ana Lemos, Cindy Gomes, Pedro Miragaia, Mário Mateus, Jorge Santos Silva

**Affiliations:** 1 Department of Pediatrics, Unidade Local de Saúde de São João, Porto, PRT; 2 Department of Pediatrics, Unidade Local de Saúde do Médio Tejo, Médio Tejo, PRT; 3 Department of Neonatology, Unidade Local de Saúde de São João, Porto, PRT

**Keywords:** congenital sucking blister, differential diagnosis, lip lesion, neonatal skin lesion, newborn

## Abstract

Congenital sucking blisters are neonatal lesions caused by intrauterine sucking activity. We report the case of a term female newborn presenting at birth with an isolated lower-lip lesion, an exceptionally rare location for this condition. The lesion evolved into a vesicle and resolved spontaneously without treatment, supporting the clinical diagnosis of a congenital sucking blister. This case highlights the importance of considering this entity in the differential diagnosis of congenital lip lesions to avoid unnecessary investigations and interventions.

## Introduction

Congenital sucking blisters are vesiculobullous lesions resulting from repetitive fetal sucking during late gestation. They occur in approximately 1 in 250 newborns and typically present at birth as flaccid, serous-filled bullae involving the thumb, fingers, hand, wrist, or forearm [1-4].

Diagnosis is primarily clinical, based on their characteristic appearance in otherwise healthy newborns, and no treatment is required because the lesions resolve spontaneously within days to weeks [1-5]. Although they may mimic infectious, inherited, or traumatic blistering disorders, accurate recognition is important to avoid unnecessary investigations, inappropriate treatment, and parental anxiety [1-6].

While congenital sucking blisters most often affect the upper extremities, involvement of the lip is exceptionally uncommon and has only rarely been reported. This unusual location may pose a diagnostic challenge because clinicians are less likely to recognize congenital sucking blisters at this site [1-6].

The following case illustrates this uncommon presentation involving the lower lip. The manuscript was prepared in accordance with the CARE reporting guidelines [7].

## Case presentation

We report the case of a term female newborn, born to healthy, non-consanguineous parents. The pregnancy was adequately monitored and uneventful. Maternal serological screening showed no evidence of infectious disease, prenatal ultrasound examinations revealed no abnormalities, and screening for Group B *Streptococcus* was negative. Delivery occurred via an uncomplicated vaginal birth. The mother had no genital lesions. Apgar scores were 9, 10, and 10 at 1, 5, and 10 minutes, respectively, and no neonatal resuscitation was required.

At birth, a solitary, firm lesion measuring less than 1 cm in diameter was observed on the lower lip. The lesion had a yellowish-white appearance, well-defined borders, and no surrounding erythema, edema, warmth, tenderness, or other signs of inflammation (Figure 1). Despite the absence of inflammatory features, the lesion raised diagnostic uncertainty due to its firmness and atypical appearance, with differential diagnoses including infectious, blistering, and even neoplastic conditions. However, the newborn remained clinically well, with good muscle tone and no systemic signs, including rash, additional lesions, vomiting, fever, or thermal instability. Given the stable clinical condition, a conservative approach was adopted, and no further diagnostic workup was performed at that time.

**Figure 1:**
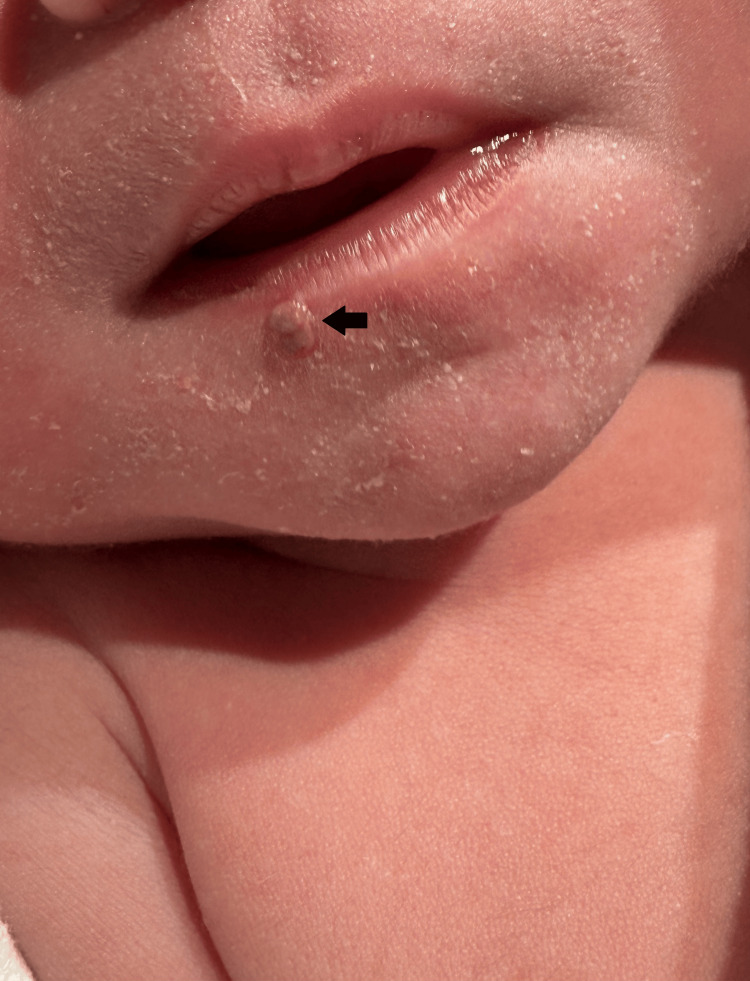
Solitary, firm yellowish-whitish lesion (arrowhead) on the lower lip observed immediately at birth

Over the following two days, the lesion evolved into a tense, well-circumscribed vesicle containing yellowish serous fluid, without surrounding erythema or other signs of inflammation (Figure 2). The newborn remained clinically well, breastfed effectively without difficulties, and had an otherwise unremarkable oral examination, with no additional mucosal lesions or other systemic abnormalities. In the absence of additional lesions or signs suggestive of infection, no further diagnostic investigations were undertaken. The infant was discharged with outpatient follow-up arranged.

**Figure 2:**
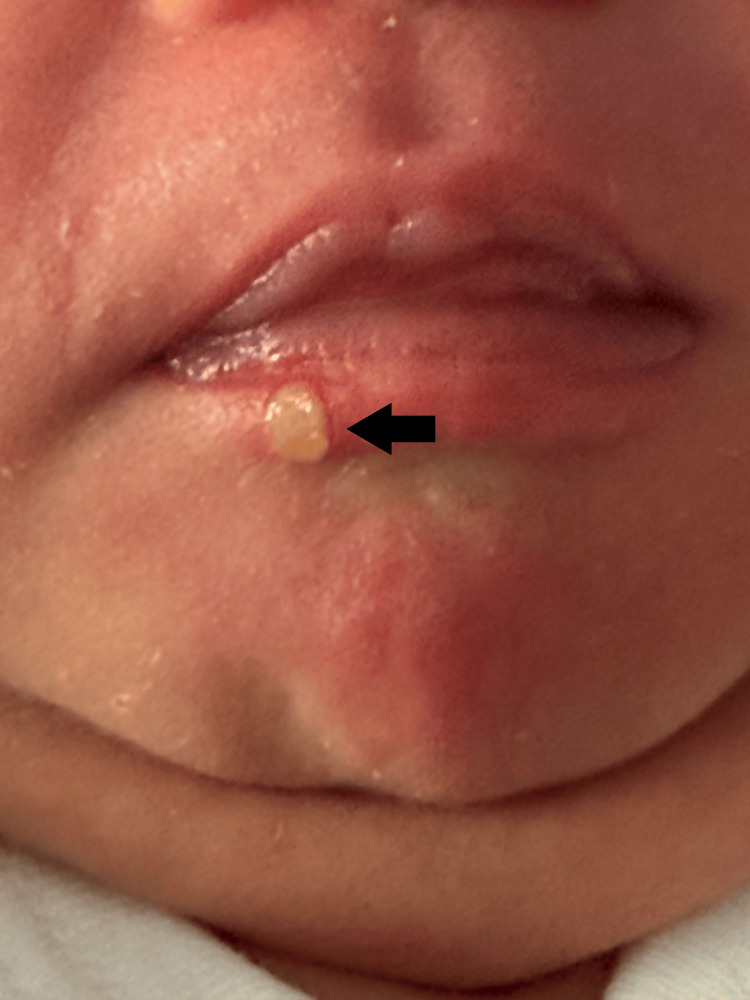
Evolution of the labial lesion into a prominent vesicle filled with yellowish serous fluid (arrowhead) on days 2-3 of life

At the one-month follow-up visit, complete spontaneous resolution of the lesion had occurred, and no residual scarring or functional impairment was noted (Figure 3). The baby demonstrated appropriate growth parameters and normal overall development.

**Figure 3:**
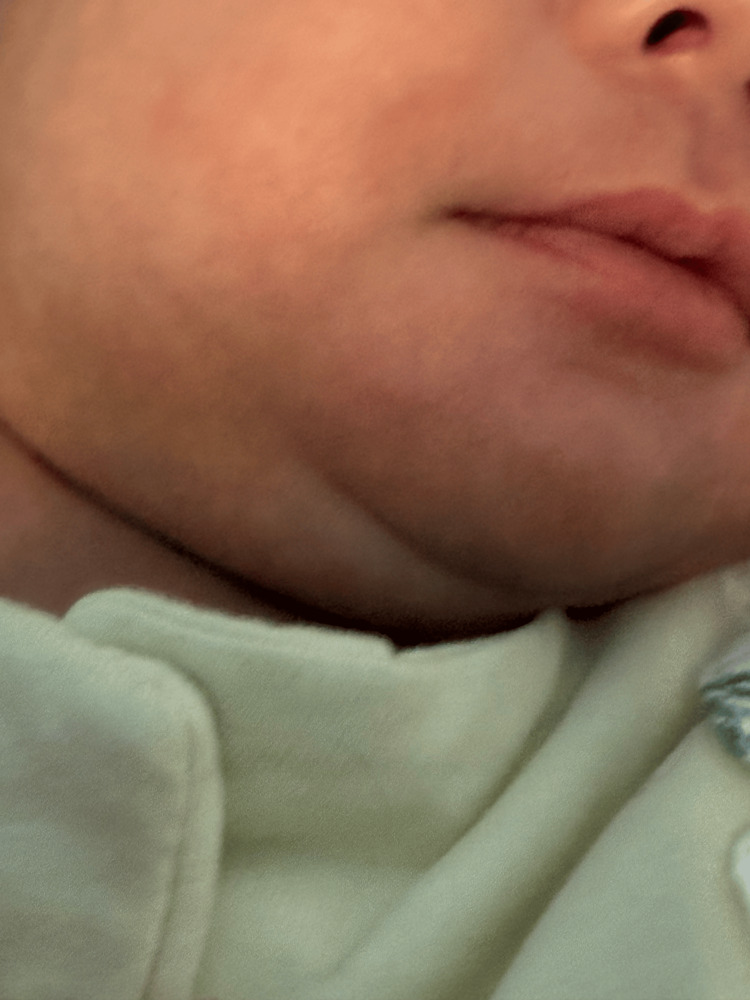
Complete spontaneous resolution of the lesion on the lower lip at one month of age

## Discussion

Cutaneous lesions in the neonatal period encompass a broad spectrum of conditions, ranging from self-limiting lesions to manifestations of serious infectious, genetic, or autoimmune disorders. In this setting, careful clinical assessment is essential to establish the correct diagnosis and guide appropriate management.

The diagnosis of congenital sucking blister is usually clinical and is based on the characteristic morphology of the lesion, its evolution, and the absence of systemic illness [1,2,5,6]. In the present case, diagnosis was particularly challenging because the lesion involved the lower lip, an exceptionally uncommon location, and initially appeared as a firm nodule before evolving into a typical fluid-filled vesicle.

This atypical presentation broadened the differential diagnosis to include traumatic lesions, congenital herpes simplex virus (HSV) infection, congenital varicella syndrome, bullous impetigo, epidermolysis bullosa, and other vesiculobullous disorders [3,5,6]. However, several clinical features argued against these conditions. The lesion was already present at birth, remained isolated, and was not associated with fever, feeding difficulties, mucosal involvement, or other systemic manifestations. Maternal infectious screening was unremarkable, and the lesion evolved spontaneously before resolving completely without treatment or scarring.

The timing of presentation was particularly helpful in excluding neonatal HSV infection. Intrapartum-acquired neonatal HSV, the most common form of neonatal herpes, usually presents between 5 and 14 days of life, whereas congenital HSV infection is rare and is typically associated with multiple skin lesions and systemic abnormalities. Likewise, congenital varicella syndrome is generally accompanied by characteristic cicatricial skin lesions and other congenital anomalies rather than an isolated labial lesion. Given the reassuring clinical findings and the characteristic evolution, no virological or cytological investigations were performed, and conservative observation was considered appropriate.

The mechanism underlying the unusual labial location remains speculative. Partial inversion of the lower lip during fetal life may have permitted repetitive suction at this site, possibly facilitated by contact with the fingers, hand, or wrist, and by fetal positioning within the uterus.

To our knowledge, congenital sucking blisters involving the lip have been reported only rarely. This case expands the spectrum of clinical presentations and highlights that congenital sucking blister should be considered in the differential diagnosis of isolated congenital lip lesions in otherwise healthy newborns. Recognition of this rare presentation may prevent unnecessary investigations, antimicrobial treatment, and parental anxiety while supporting appropriate conservative management.

## Conclusions

This case describes a rare lower-lip lesion clinically consistent with a congenital sucking blister. The diagnosis was supported by its characteristic clinical course and spontaneous resolution without treatment. Awareness of this rare localization may broaden the recognized clinical spectrum of congenital sucking blisters and assist clinicians in considering this entity in the differential diagnosis of congenital lip lesions. Careful clinical assessment and observation can help avoid unnecessary investigations and interventions.
